# Sex differences in sleep architecture among patients with severe mental disorders: a polysomnographic study

**DOI:** 10.3389/fpsyt.2026.1876676

**Published:** 2026-10-07

**Authors:** Zhimin Lan, Wei Lin, Meng Wang, Xiaofang Chen, Meihong Xiu, Anle Pan

**Affiliations:** 1 Peking University HuiLongGuan Clinical Medical School, Beijing Huilongguan Hospital, Capital Medical University, Beijing, China; 2 Qingdao Mental Health Center, Qingdao, China; 3 The Second People’s Hospital of Lishui, Zhejiang Provincial Clinical Research Center for Mental Disorders, Wenzhou, China

**Keywords:** bipolar disorder, major depressive disorder, polysomnographic, schizophrenia, sex difference, sleep

## Abstract

**Background:**

Sleep disturbances are common in severe mental illness, including schizophrenia, bipolar disorder, and major depressive disorder. Yet sex-specific sleep patterns across schizophrenia, bipolar disorder, and major depressive disorder remain poorly characterized.

**Methods:**

In this cross-sectional study, we compared overnight polysomnography between 372 first-episode patients (aged 18-45) (93 schizophrenia, 181 major depressive disorder, 98 bipolar disorder). All participants underwent a single-night 10-channel polysomnography.

**Results:**

After controlling for age, illness duration, BMI, and medications, women achieved significantly longer total sleep time (median 449.3 vs 420.3 min; p <0.001), time in bed (543.0 vs 525.5 min; p = 0.03), and higher sleep efficiency (83.3% vs 82.3%; p = 0.004) than men. Exploratory *post-hoc* analyses revealed potential sex-related differences in major depressive disorder and bipolar disorder (all p < 0.05, uncorrected for multiple comparisons). However, these findings did not survive correction for multiple comparisons, indicating that the observed differences should be interpreted with caution as preliminary.

**Conclusions:**

This study found that biological sex modulated sleep macro-architecture in severe mental illness, with women exhibiting longer sleep duration and higher sleep efficiency relative to men. Although the cross-diagnosis comparison revealed only a marginally significant sex-by-diagnosis interaction that did not persist after multiple testing correction, these exploratory subgroup findings suggest potential nuanced variations in sleep architecture. Consequently, future studies with larger cohorts are warranted to evaluate whether sex-stratified approaches could refine clinical sleep interventions across psychiatric conditions.

## 1 Introduction

Sleep is a dynamic interplay of physiologic and behavioral processes, and it constitutes a fundamental pillar of cognitive, emotional, and physical health (1). Decades of studies have provided evidence for a strong association between sleep alterations and mental disorders (2). Difficulties initiating, maintaining, and timing sleep are common complaints among patients with severe mental illness (SMI) and are linked to disability, distress, and poorer quality of life. These alterations are not merely common symptoms (3), and they are also key risk factors for both the onset and persistence of psychiatric disorders (4–7). Accurate assessment and understanding of sleep abnormalities in SMI are therefore essential for comprehensive clinical management.

SMI ranks as the leading contributor to the global disease burden of mental disorders and drives substantial disability, premature mortality, and profound societal costs (8). A robust literature reports that different SMIs, such as schizophrenia (SCH), bipolar disorder (BD), and major depressive disorder (MDD), exhibit distinct sleep patterns. For example, SCH patients exhibited difficulty initiating and maintaining sleep, along with episodes of excessive sleep, frequent nightmares, and irregular sleep-wake patterns (9–11). In particular, objective measurements strongly support these subjective reports. Actigraphy studies show lower sleep efficiency (SE), greater sleep fragmentation, and shorter total sleep time in patients with SCH than in healthy controls (12). Polysomnography studies reveal lower sleep time and a range of disturbances in sleep architecture in patients with SCH compared with controls (13). Crucially, clinical observations point to the fact that such disturbances often emerge before the first psychotic episode (14–17). For patients with MDD, an epidemiological study reported that 92% experience at least one type of sleep complaint, with 85% reporting insomnia, 48% hypersomnia, and 30% both insomnia and hypersomnia (18). In MDD, polysomnography revealed disrupted sleep continuity and altered REM architecture, patterns that mirror those observed in primary insomnia (19, 20). For BD, reduced need for sleep is a hallmark of mania, whereas insomnia and hypersomnia typify bipolar depression. Meta-analyses of actigraphy data further show that patients with inter-episode BD exhibit prolonged sleep-onset latency, extended wake after sleep onset, longer total sleep time, and consequent reductions in SE relative to controls (21).

Despite this wealth of evidence, a crucial and often overlooked variable is sex. In healthy populations, sleep architecture (e.g., slow-wave sleep and REM sleep) shows marked sex differences. Within psychiatric disorders, sex differences are evident in disease risk, age at onset, symptom presentation, and treatment response (22–25). Yet, most sleep studies in SMI have either ignored sex or treated it merely as a confounding covariate. Consequently, it remains unclear to what extent the sleep abnormalities observed in SMI patients are influenced by sex and whether sex-specific differences were consistent across different diagnoses (SCH, BD, MDD). Systematically examining the sex-specific sleep characteristics within each diagnostic group is essential for understanding the heterogeneity of SMI, but this question remains largely unexplored.

Normal human sleep consists of alternating cycles of rapid eye movement (REM) and non-rapid eye movement (NREM) sleep, yet this architecture is frequently disrupted in psychiatric populations. Although PSG provides an objective means of quantifying disturbances in both sleep continuity and structure, how sex influences these parameters across distinct SMIs remains insufficiently characterized. Distinct neurobiological mechanisms may drive disorder-specific sex dimorphisms in sleep architecture. Specifically, the potent neuroprotective role of estrogen in SCH (26, 27) and the prominent endocrine and HPA axis dysregulation in female MDD (28) suggest a high susceptibility to sex-specific sleep alterations in these two disorders. Conversely, BD is fundamentally driven by pervasive, core circadian rhythm disruptions (29, 30) that may operate across sexes regardless of endocrine variation.

To address this gap, the present study utilized overnight PSG to systematically examine sex differences in sleep parameters within a cohort encompassing the three SMIs: SCH, BD, and MDD. We hypothesized that significant sex differences would persist after controlling for potential confounders such as age, BMI, illness duration, and medications. In addition, we further examined whether these sex effects varied across three SMIs as an exploratory aim.

## 2 Methods

### 2.1 Study population

The present study is a non-interventional, cross-sectional study of individuals with SMI conducted at three hospitals. A total of 372 first-episode patients (93 patients with the diagnosis of SCH, 181 with MDD, and 98 with BD) were recruited from 2016.10 to 2024.12. The age range of this study population was 18-45 years, and diagnoses received before and after this age span were not included. The diagnoses were made by experienced psychiatrists using the Structured Clinical Interview of the Diagnostic and Statistical Manual of Mental Disorders (SCID), 5^th^ Edition. Participants were interviewed and evaluated during their inpatient stay.

A total of 900 patients were screened. Members of the research group approached eligible patients, explained the study’s aims and procedures, and issued a written invitation for them to participate. Individuals who expressed interest were invited to a quiet interview room where trained staff administered the SCID, Axis I Disorders to confirm a diagnosis of SCH, MDD, or BD. Personality disorders and Axis II rule-outs were evaluated using the Structured Clinical Interview for DSM-5 Personality Disorders (SCID-II/SCID-5-PD). To ensure diagnostic stability and rule out transient psychotic symptoms, affective psychoses, or emerging comorbidities, all initially screened patients underwent a mandatory diagnostic re-evaluation six months after the baseline assessment. Sixteen patients withdrew or were lost to follow-up, and 375 patients underwent PSG recording (**Supplementary Figure 1**). Patients initially screened for MDD underwent clinical re-evaluation, during which 10 individuals changed the diagnosis of MDD, based on documented hypomanic or manic episodes. After PSG recording, three participants were excluded from the analysis due to time in bed (TIB) of less than 5 hours, and 372 patients were included in the final analysis.

The inclusion criteria included: I) patients with SCH, MDD, or BD; 2) first episode; 3) duration of illness < 5 years; and 4) Han Chinese. For BD, patients enrolled in this study were allowed to have previous depressive episodes as long as their diagnosis was other than BD and the current manic episode was the one that established their BD diagnosis. The exclusion criteria were organic brain disease, substance dependence, any severe physical illness undergoing drug treatment, any history of head trauma, comorbidity with other types of Axis I or Axis II mental disorders, any contraindication to performing a PSG monitor, pregnancy, or breastfeeding. In addition, participants underwent a rigorous screening process to rule out primary sleep disorders, including obstructive sleep apnea (OSA), periodic limb movement disorder (PLMD), and parasomnias. Among 372 patients, 40 were drug-naïve, and 332 had been on short-term psychotropic treatment (<2 weeks). Antipsychotic drugs included olanzapine (n=139), risperidone (n=40), and aripiprazole (n=38). Additionally, 71 patients received sertraline, 20 were prescribed mood stabilizers (lithium or valproate), and 219 took sleep medicines. Among the 98 patients with BD, 80 were diagnosed with BD type I (BD-I) and 18 with BD type II (BD-II).

The Ethics Committee of the hospital approved the study (Ethics No.: Y27-07). After receiving a full explanation of the protocol, patients gave written informed consent.

### 2.2 PSG acquisition and sleep structure analysis

Sleep recordings were carried out using an overnight PSG as described in a previous study (31). In brief, PSG began at each participant’s habitual bedtime and continued until spontaneous morning awakening. The sleep laboratory is maintained at an appropriate temperature and humidity. Subjects were advised to keep their usual sleep schedules, with minor adjustments if necessary. Patients with a total TIB of less than 5 hours were excluded from the PSG analysis. PSG recordings were conducted shortly after hospital admission (within 1-5 days of admission). At the time of recording, patients were in the acute phase of illness.

Whole-night PSG was performed and scored according to the 2007 AASM guidelines (32). It included wakefulness, N1, N2, N3, and REM phase. The reference values for adult sleep macrostructure (as percentages of total sleep time) in healthy control subjects are approximately 2-5% for N1, 45-55% for N2, 15-20% for N3, and 20-25% for REM. Continuous respiratory monitoring, including nasal pressure airflow, thoracoabdominal respiratory effort, and pulse oximetry, was recorded, and the apnea-hypopnea index (AHI) was calculated according to the American Academy of Sleep Medicine (AASM) scoring rules.

### 2.3 Statistical analysis

All analyses were conducted in SPSS. The difference was considered significant with p<0.05 (two-tailed).

The Kolmogorov-Smirnov one-sample test was used to assess the normality of the continuous variables. Categorical variables are summarised as percentages and group frequencies, with inter-group differences evaluated by *χ*² tests. Considering that most variables, in particular sleep parameters, were not normally distributed, continuous variables were described by median and IQR. Chi-square tests and the non-parametric Mann-Whitney U test were performed as appropriate to compare the sleep parameters between male and female groups. To correct for multiple comparisons, a Bonferroni correction was applied across the 11 sleep variables, with the significance threshold set at α = 0.05/11 ≈ 0.0045. Thus, a two-sided p < 0.0045 was considered statistically significant.

An *a priori* sample size calculation was performed using G*Power software for two independent groups (non-parametric Wilcoxon-Mann-Whitney test). Based on preliminary findings (33), we assumed a moderate effect size of Cohen’s d = 0.37. Setting two-tailed α = 0.05 and a desired statistical power of 0.80, the analysis indicated that a total sample size of 250 would provide adequate power to detect statistically significant differences between male and female patients.

To comprehensively explore the association between sex and sleep parameters across the distribution (25th, 50th, and 75th percentiles) in all patients with SMI, we performed quantile regression analysis using the R (v4.5.2) package quantreg, with 1, 000 bootstrap replications. To test whether sex differences varied across diagnostic subgroups, interaction models including the interaction term (Sex and Diagnosis) were primarily established, with adjustments for age, illness duration, BMI, and medications. Sex was coded as sex1 = male and sex2 = female, with male as the reference group in regression analyses. Quantile regression coefficients (β) were estimated for each quantile, and the significance of the interaction terms was assessed using Wald tests with bootstrap resampling (1, 000 replicates). All visualizations were generated using the ggplot2 package (v3.5.1).

## 3 Results

### 3.1 Demographic and clinical characteristics in patients

No significant difference was observed in age, duration of illness, and educational levels between male and female patients (all p>0.05) (**Table 1**). There was no significant difference in the diagnosis of SMI between men and women. Onset age and BMI were different between male and female patients (F = 5.0, p=0.03; F = 8.6, p=0.004). Spearman correlation analysis showed significant associations between age, onset age, illness duration, and sleep parameters in all patients.

**Table 1:** Comparisons of demographic and clinical characteristics between male and female patients with mental disorders.

Characteristics	All patients (n=372)	Males (n=146)	Females (n=226)	*F (p)*
Age (ys)	27.8 ± 8.1	26.8 ± 8.0	28.4 ± 8.1	3.7 (0.06)
Onset age (ys)	25.1 ± 8.1	24.1 ± 8.0	25.8 ± 8.1	4.0 (0.046)
Diagnosis of SMI, n (%)				0.4 (0.82)
SCH	93	39	54	
BD	98	38	60	
MDD	181	69	112	
Education levels, n (%)				4.8 (0.19)
Primary school	22	7	15	
Junior school	46	12	34	
Senior school	283	119	164	
Above	21	8	13	
BMI	23.7 ± 4.6	24.5 ± 4.3	23.1 ± 4.7	8.6 (0.004)

SMI, severe mental illness; SCH, schizophrenia; BD, bipolar disorder; MDD, major depressive disorder; BMI, body mass index.

### 3.2 Comparisons of sleep parameters between male and female patients

We found a significant sex difference in TIB (525.5 vs 543.0, Z = 2.2, p=0.03, Cohen’s *d* = 0.23) (**Figure 1**), total sleep time (TST) (420.3 vs 449.3, Z = 3.6, p<0.001, Cohen’s *d* = 0.38) (**Figure 2**) and SE (82.3% vs 83.3%, Z = 2.9, p=0.004, Cohen’s d=0.31) (**Table 2**) (**Figure 3**). Female patients slept longer and had a median of 29.0 minutes more than men, equivalent to 6.9% of the TST. Females had a better SE and a median of 1.0% more than men, equivalent to 1.2% of the SE. In addition, females spent more time in bed, with a median of 17.5 minutes longer than males, equivalent to 3.3% of the TIB. After Bonferroni corrections, the differences in TST and SE remained significant.

**Figure 1:**
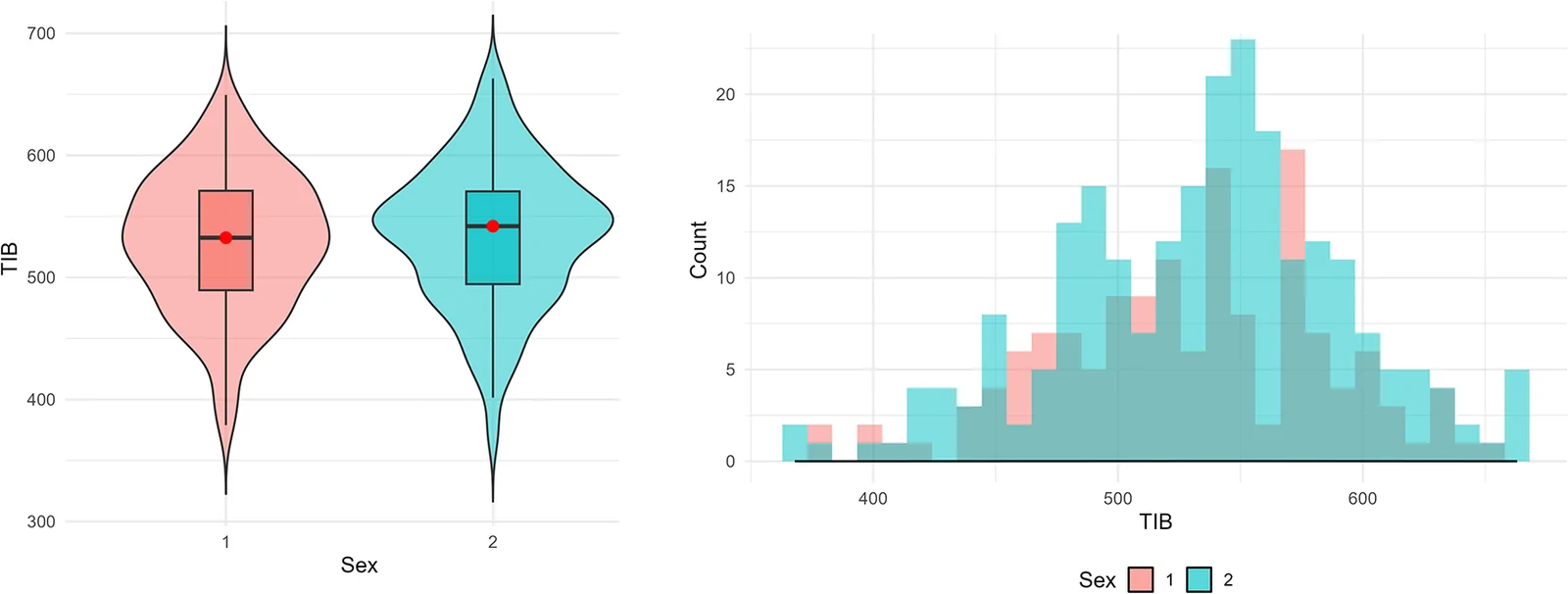
Distribution of time in bed (TIB) by Sex (left) Violin plots with overlaid box plots showing the distribution of TIB for males and females. The violin width indicates the kernel density of the data. (right) Histograms with kernel density curves illustrating the frequency distribution of TIB for each sex.

**Figure 2:**
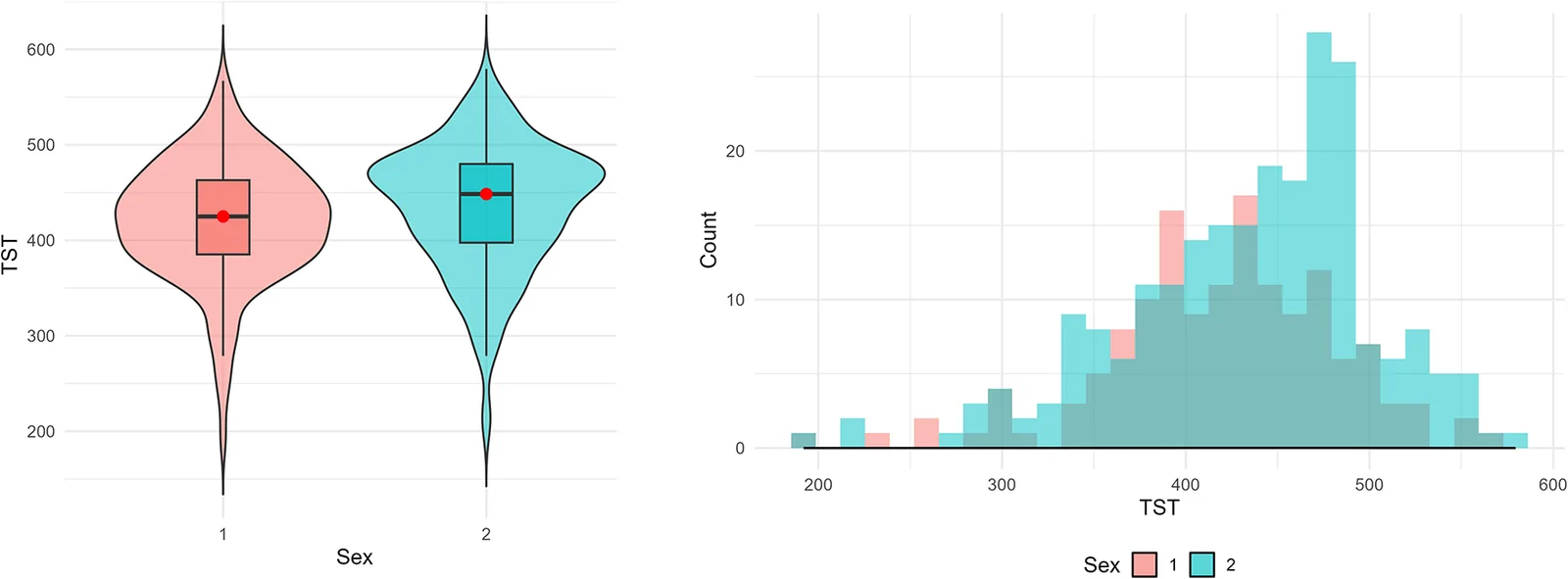
Distribution of total sleep time (TST) by Sex (left) Violin plots with overlaid box plots showing the distribution of TST for males and females. The violin width indicates the kernel density of the data. (right) Histograms with kernel density curves illustrating the frequency distribution of TST for each sex.

**Table 2:** Comparisons of sleep parameters between male and female patients.

Variables	All patients (n=372)	Males (n=146)	Females (n=226)	*Z (p)*
TST (min)	435.7 (388.5, 476.0)	420.3 (377.9, 460.4)	449.3 (398.5, 480.6)	3.6 (<0.001)
TIB (min)	537.81 (492.4, 570.0)	525.5 (481.1, 569.6)	543.0 (495.4, 572.5)	2.2 (0.03)
SE (%)	82.8 (78.7, 85.5)	82.3 (76.7, 84.7)	83.3 (79.8, 85.9)	2.9 (0.004)
WASO	14.5 (6.0, 32.0)	14.0 (5.5, 36.1)	15.0 (6.4, 28.1)	0.2 (0.85)
AT	3.0 (1.0, 5.0)	3.0 (1.0, 5.0)	2.0 (1.0, 5.3)	0.7 (0.50)
SOL	9.5 (4.5, 17.5)	10.5 (4.5, 20.1)	8.5 (4.5, 16.0)	1.2 (0.25)
REML (min)	210.5 (126.0, 297.9)	204.5 (115.5, 293.1)	216.5 (132.3, 300.0)	0.8 (0.41)
N1%	4.6 (2.4, 7.9)	4.6 (2.3, 8.6)	4.4 (2.4, 7.7)	0.5 (0.64)
N2%	81.5 (71.3, 88.6)	82.1 (71.6, 88.5)	80.6 (70.5, 88.6)	0.04 (0.97)
N3%	0 (0.0, 9.7)	1.4 (0.0, 9.8)	0 (0.0, 9.6)	0.7 (0.48)
REM%	8.5 (4.6, 13.4)	8.3 (4.4, 13.2)	9.2 (4.9, 13.7)	0.9 (0.37)

TIB, time in bed; TST, total sleep time; SE, sleep efficiency; WASO, wake after sleep onset; AT, arousal times; SOL, sleep onset latency; REML, REM sleep latency.

**Figure 3:**
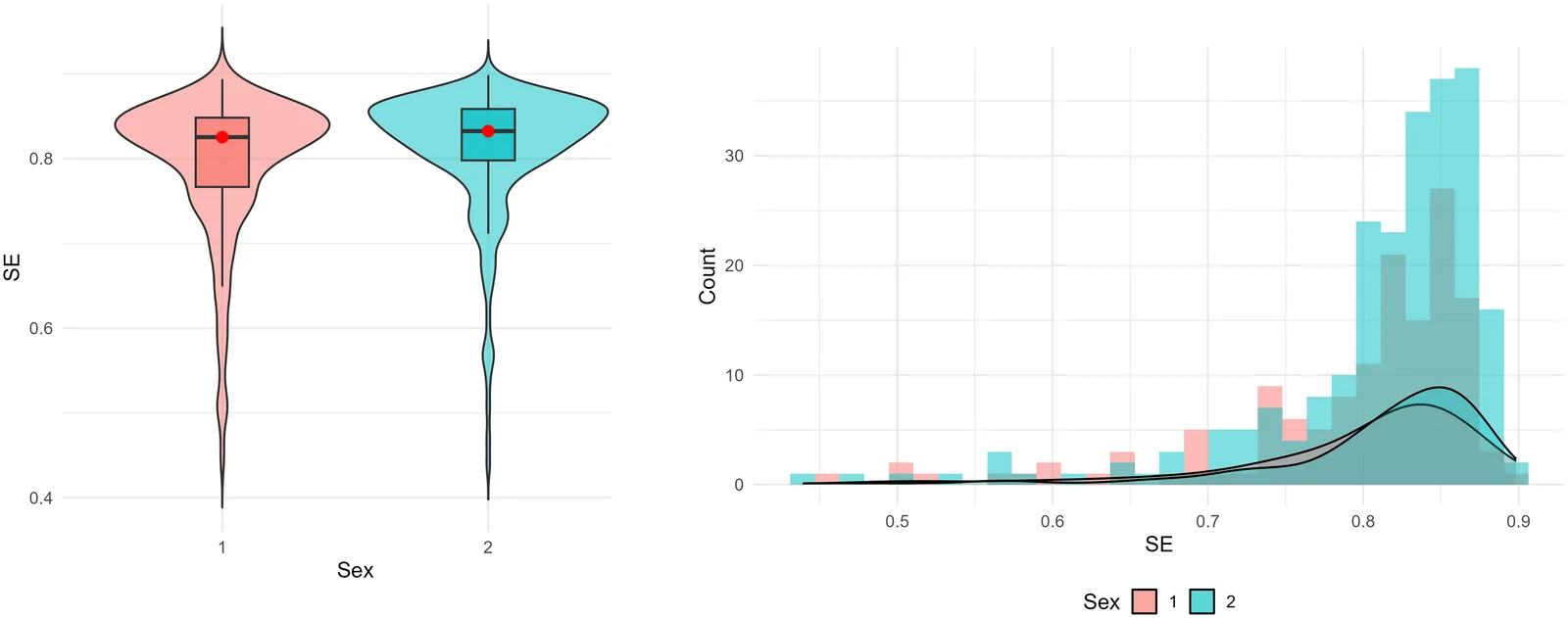
Distribution of Sleep Efficiency (SE) by Sex (left) Violin plots with overlaid box plots showing the distribution of SE for males and females. The box plot displays the median (red point), and interquartile range (IQR, box). The violin width indicates the kernel density of the data. (right) Histograms with kernel density curves illustrating the frequency distribution of SE for each sex.

We further performed quantile regression analysis to verify the associations after controlling for age, illness duration, BMI, and medications. Since age was significantly associated with onset age in our study, only age was included in the models. To comprehensively account for pharmacological influences, we re-encoded medication use into a novel four-level categorical variable: group 0 (medication-free/reference group), group 1 (antipsychotic monotherapy), group 2 (sedative/hypnotic monotherapy), and group 3 (combined use). This classification was subsequently included as a covariate in the regression analyses. The results showed that sex (sex2 vs sex1) significantly predicted TST across all quantiles examined. At the 25th percentile (lower sleep duration), sex2 had a TST that was 22.98 minutes longer than sex1 (β = 22.98, t = 3.13, p = 0.002). At the 50th percentile (median sleep duration), sex2 had a TST that was 31.88 minutes longer than sex1 (β = 31.88, t = 4.25, p < 0.001). At the 75th percentile (higher sleep duration), sex2 had a TST that was 19.55 minutes longer than sex1 (β = 19.55, t = 2.68, p = 0.008).

Similarly, sex (sex2 vs sex1) significantly predicted SE at the median and higher levels of sleep efficiency, with a marginally significant effect at the lower level. At the 25th percentile (lower sleep efficiency), sex2 had a SE that was 0.016 higher than sex1 (β = 0.016, t = 1.26, p = 0.210). At the 50th percentile (median sleep efficiency), sex2 had a SE that was 0.013 higher than sex1 (β = 0.013, t = 2.06, p = 0.04). At the 75th percentile (higher sleep efficiency), sex2 had a SE that was 0.012 higher than sex1 (β = 0.012, t = 3.29, p = 0.001).

However, sex differences in TIB varied across the distribution of sleep duration. At the 25th percentile, women had a marginally longer TIB than men (β = 20.02, t = 1.83, p = 0.069). At the 50th percentile, the sex difference was significant, with women having 16.28 minutes longer TIB than men (β = 16.28, t = 2.55, p = 0.011). However, at the 75th percentile, the sex difference was not significant (β = -0.37, t = -0.04, p = 0.965).

Three participants were excluded from the final analysis because they had fewer than 5 hours of TIB. We then performed the sensitivity analysis to examine whether this introduced selection bias and found that the results remained consistent regardless of whether these 3 participants were included or excluded.

### 3.3 Sex differences in sleep parameters in the SCH, MDD, and BD groups

We further analyzed the sleep parameters across the three diagnostic groups within SMI between males and females, and tested whether these differences varied by diagnosis. When exploring these sex differences exploratorily within each diagnostic group, significant differences in TST were found in the MDD and BD groups (all p<0.05), in TIB for the BD group (Z = 2.7, p=0.007), and in SE for the MDD and BD groups (all p<0.05) (**Table 3**, **Figure 4**). None of the differences passed the Bonferroni correction.

**Table 3:** Comparisons of sleep parameters between males and females in SCH, MDD, and BD.

	MDD group Males (n=69) vs Females (n=112)	SCH group Males (n=54) vs Females (n=39)	BD group Males (n=38) vs Females (n=60)
TST (min) Z (p)	425.0 (389.8, 462.5)vs452.5 (410.8, 481.9) 2.6 (0.01)	415.0 (366.0, 462.5)vs431.3 (360.1, 474.4) 0.8 (0.43)	406.3 (369.8, 447.1)vs454.8 (406.1, 484.4) 2.8 (0.006)
TIB (min) Z (p)	536.0 (491, 569.8)vs542.3 (504.0, 578.9) 1.2 (0.23)	524.5 (488.0, 573.0)vs541.8 (482.9, 565.0) 0.1 (0.91)	505.0 (460.1, 545.9)vs545.3 (495.1, 574.6) 2.7 (0.007)
SE (%) Z (p)	82.3 (77.9, 84.9)vs83.5 (80.2, 86.0) 2.7 (0.007)	83.2 (70.0, 85.3)vs80.8 (76.6, 84.5) 0.3 (0.79)	81.9 (78.6, 84.4)vs84.6 (81.4, 86.4) 2.7 (0.007)
WASO Z (p)	14.0 (6.5, 38.0)vs14.5 (6.5, 27.0) 0.9 (0.39)	14.0 (4.5, 52.0)vs20.5 (9.0, 48.6) 0.9 (0.35)	15.0 (3.9, 32.0)vs12.0 (3.6, 21.0) 0.5 (0.62)
AT Z (p)	3.0 (1.0, 5.0)vs2.5 (1.0, 6.0) 0.7 (0.51)	3.0 (1.0, 6.0)vs2.0 (1.0, 5.0) 0.4 (0.68)	3.0 (1.0, 4.3)vs2.5 (1.0, 6.0) 0.1 (0.90)
SOL Z (p)	11.5 (6.0, 20.5)vs8.5 (5.0, 16.4) 1.7 (0.08)	8.5 (2.5, 21.0)vs11.5 (6.1, 16.8) 1.2 (0.22)	8.3 (3.5, 16.6)vs6.0 (2.6, 13.4) 1.2 (0.22)
REML (min) Z (p)	232.5 (149.3, 337.8)vs264.3 (152.2, 327.8) 0.5 (0.63)	172.5 (99.0, 288.5)vs190.3 (130.8, 275.9) 0.9 (0.38)	187.3 (93.6, 249.1)vs175.8 (113.4, 236.5) 0.004 (0.99)
N1% Z (p)	5.2 (3.1, 9.2)vs4.9 (2.7, 7.4) 1.3 (0.19)	3.0 (1.7, 6.8)vs4.0 (1.7, 7.7) 1.6 (0.12)	4.4 (1.9, 8.7)vs5.5 (2.4, 7.7) 0.8 (0.41)
N2% Z (p)	82.6 (69.7, 89.2)vs84.1 (76.2, 90.6) 1.6 (0.11)	83.9 (77.1, 88.4)vs79.4 (72.5, 87.7) 1.0 (0.33)	78.2 (68.4, 86.3)vs74.2 (66.7, 83.2) 1.3 (0.21)
N3% Z (p)	1.1 (0, 9.8)vs0 (0, 5.3) 2.3 (0.02)	0 (0, 9.6)vs0.8 (0, 10.3) 1.0 (0.30)	5.6 (0, 10.3)vs6.5 (0, 15.2) 0.8 (0.46)
REM% Z (p)	7.4 (3.9, 12.2)vs7.8 (3.6, 12.3) 0.2 (0.86)	8.5 (4.6, 12.7)vs9.4 (5.4, 12.9) 0.3 (0.77)	9.8 (4.4, 13.7)vs11.9 (7.1, 15.8) 1.4 (0.15)

**Figure 4:**
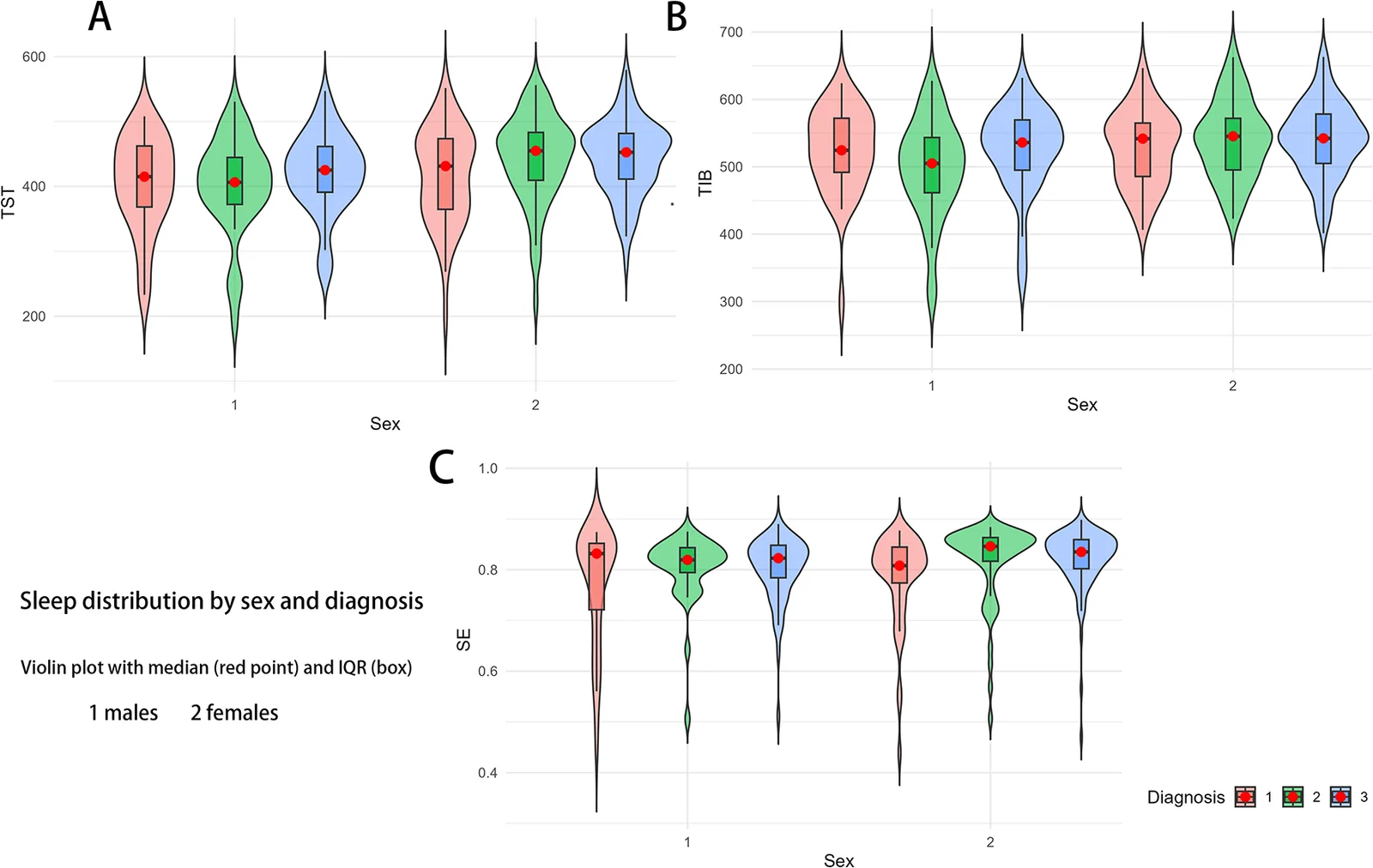
Distribution of sleep parameters by sex and diagnosis Violin plots display the distribution density of **(A)** TST (interaction effect: β=27.2, p=0.25); **(B)** TIB (interaction effect: β=46.1, p=0.06); **(C)** SE (interaction effect: β=0.04, p=0.06). The embedded box plots represented the median (red point) and interquartile range (IQR).

However, because the interaction effects between sex and diagnosis on TST, TIB, and SE were not statistically significant (β=27.2, p=0.25; β=46.1, p=0.06; and β=0.04, p=0.06), these subgroup-specific results should be interpreted with caution and were presented as descriptive findings rather than definitive group-by-sex differences.

## 4 Discussion

We examined overnight polysomnography in 372 patients with SCH, BD, or MDD and documented sex-specific sleep macro-architecture across the three most common SMIs. After controlling for age, illness duration, BMI, and medications, female patients slept longer and achieved higher SE than males. *Post-hoc* analyses showed that sex differences in sleep parameters were present across all three diagnostic groups. However, only the MDD group remained a significant sex difference, while the effects in the SCH and BD groups did not survive correction. These results indicate that sex modulated sleep continuity in SMI, but the statistical robustness of this effect varies by diagnostic category.

Our findings align with previous studies showing that female patients with SMI consistently exhibited longer total sleep time, time in bed, and higher SE compared to male patients. Notably, the magnitude of the sex-specific elevation in sleep parameters observed in our study, specifically 29 minutes longer TST, 17.5 minutes longer TIB, and 1.0% higher SE, was markedly pronounced, exceeding the effect sizes reported in adult healthy cohorts in a previous review (34). However, that review primarily relied on self-reported time-use surveys, which were not directly comparable to our objective PSG measurements. As our study did not include a healthy control group, direct statistical comparisons remained unfeasible. Therefore, the true disparity in sleep parameters between SMI and healthy individuals, as well as the extent to which SMI modulated this sex difference, remained uncertain. Taken together, this review and our findings confirmed that distinct sex-specific differences in sleep parameters were present.

Furthermore, interaction analyses and stratified comparisons revealed no statistically significant sex differences in sleep architecture within any of the three diagnostic groups (SCH, BD, and MDD) after correction for multiple testing. This finding aligned with a growing body of evidence documenting sex differences in sleep architecture among both healthy populations and psychiatric patients (10–12, 35). We speculate that these sex-dependent sleep patterns may be related to several mechanisms at different levels. First, sex hormones, particularly estrogen and progesterone, are known to modulate sleep-wake regulation, circadian rhythms, and REM sleep pressure (9). Fluctuations in these hormones across the menstrual cycle, as well as their absolute levels, may differentially affect sleep continuity and architecture in women compared with men, even in the presence of psychiatric illness. Second, the neurobiological underpinnings of the three disorders-such as hyperactivity of the HPA axis, altered monoaminergic neurotransmission, and inflammatory changes-may interact with sex-specific genetic and epigenetic factors, resulting in divergent sleep phenotypes between sexes (36–39). Third, psychological factors, including nocturnal cognitive arousal and emotion-focused rumination, have been implicated in sleep abnormalities in mental disorders (31). We acknowledge that these interpretations remained speculative, as the current investigation was phenomenological. The specific pathways and their relative contributions to the observed sex differences warranted confirmation in larger, pharmacologically controlled, and longitudinal samples. Furthermore, potential confounding factors such as menopausal status, phase of menstrual cycle, and oral contraceptive use were not recorded, which may have introduced additional variability.

Our findings revealed a median N3 sleep percentage of 0% and REM sleep percentage of 8.5%, which were lower than the normative reference values reported in previous meta-analyses (33). This discrepancy may be primarily attributable to a pronounced first-night effect, as in-laboratory polysomnography can significantly disrupt sleep architecture, particularly reducing slow-wave sleep and REM sleep duration. Furthermore, the normative values reported by Boulos et al. (33) reflected baseline sleep parameters derived from healthy controls without confounding medical or sleep conditions; in contrast, our study sample comprised patients experiencing an acute exacerbation of clinical symptoms, whose acute disease state, associated physical discomfort, emotional anxiety, and physiological hyperarousal may have further suppressed deep and REM sleep. While these values reflected the sleep parameters of our specific study sample during overnight laboratory monitoring, caution should be exercised when comparing these architecture metrics with general population norms derived from healthy individuals.

There were several strengths and limitations in the present study. Strengths included the large inpatient sample, blinded PSG scoring, and rigorous covariate control. Several limitations deserved emphasis: (1) The cross-sectional design precluded causal inferences about whether sex differences precede or follow symptom changes; (2) Medication was not discontinued, so sedative effects of antipsychotics or antidepressants may have interacted with sex; (3) The restricted age range of participants (18-45 years) limited generalisability to older adults; (4) We did not assess menstrual phase or hormonal contraception. Given the central role of estrogen and progesterone in modulating sleep architecture and REM pressure discussed above, unmeasured hormonal variability may have confounded our sex-based comparisons. The observed sex differences in sleep parameters may partly reflect heterogeneity in reproductive endocrine status rather than pure biological sex effects or diagnosis-specific mechanisms. Future studies should stratify analyses by menstrual cycle phase or directly assay circulating estradiol and progesterone to disentangle endocrine influences from illness-related sex differences. (5) The sample was recruited from inpatient settings, which may limit the generalizability of our findings to outpatients or community-dwelling individuals with mental disorders. Inpatient populations typically represented individuals with greater illness acuity, more severe symptomatology, or poorer functional status, thereby restricting the range of psychopathology. (6) We did not collect medication dose, smoking, and alcohol/caffeine use, and were therefore unable to adjust for these factors in the regression analysis. In addition, illness severity (PANSS/HAMD/YMRS) was also not assessed or included as a covariate. Future studies should incorporate comprehensive medication doses, substance use, and standardized symptom severity ratings to clarify these associations. (7) The absence of baseline data for the 16 eligible participants who were not enrolled (3.3% attrition). Although this attrition rate was low, we could not exclude the possibility that non-respondents differed from enrolled participants in unmeasured characteristics such as illness severity or sleep disturbance severity. (8) All participants underwent a single-night polysomnographic assessment without a prior adaptation night. It is well-established that the first night in a sleep laboratory produced an artificial sleep environment that could alter sleep architecture. Consequently, the sleep parameters reported in this study may not fully reflect participants’ habitual sleep quality under naturalistic conditions. (9) Although sleep medication use was statistically controlled in our multivariable analyses, the high prevalence of sleep medication use in our sample (59%) remains a key limitation. In clinical practice, patients with acute SCH and MDD, in particular BD, frequently required pharmacological intervention to achieve sufficient compliance and complete overnight PSG recordings. Despite analytical adjustments, residual confounding from varied medication types and individual metabolic responses cannot be fully ruled out, which may have masked the characteristic sleep disturbance pattern in participants. (10) Our MDD sample may have included patients with undiagnosed BD who had not yet experienced a manic episode at enrollment and 6-month confirmation. This potential diagnostic overlap could have minimized the true differences in sleep architecture between the three groups, and longitudinal follow-up studies are needed to confirm the specificity of our findings. (11) The episode polarity (i.e., manic, hypomanic, depressive, or mixed) at the time of the PSG recording was not systematically documented. Because PSG was conducted during the acute phase (within 1–5 days of admission), sleep architecture, particularly TST, SE, and slow-wave sleep, may vary substantially depending on whether a patient is experiencing mania or depression. Furthermore, if there was an uneven distribution of episode polarity between male and female participants, this unmeasured confounding factor could potentially account for or influence the observed sex differences within the BD subgroup. Future studies should explicitly record and adjust for acute mood state polarity to better isolate sex-specific sleep alterations in BD.

## 5 Conclusions

Using overnight PSG in a large inpatient sample, we provided further evidence that sex differences in sleep were present across all three major SMIs. After rigorous adjustment for demographic and clinical confounders, exploratory *post-hoc* analyses suggested a potential female advantage in sleep duration and efficiency among patients with BD and MDD, but these were not confirmed after adjustment. Our preliminary findings did not support strong diagnostic heterogeneity in sleep-related sexual dimorphism. Given the cross-sectional and descriptive nature of our study, these findings did not establish a causal relationship between biological sex and disorder-specific sleep architecture. Future longitudinal studies were warranted to elucidate the underlying circadian, neuroendocrine, or disorder-specific mechanisms so that sex-stratified strategies may be developed to optimize sleep and ultimately improve clinical outcomes in patients with SMIs.

## Data Availability

The raw data supporting the conclusions of this article will be made available by the authors, without undue reservation.

## References

[1] Wulff K Gatti S Wettstein JG Foster RG . Sleep and circadian rhythm disruption in psychiatric and neurodegenerative disease. Nat Rev Neurosci. (2010) 11:589–99. doi: 10.1038/nrn2868 20631712

[2] Freeman D Sheaves B Waite F Harvey AG Harrison PJ . Sleep disturbance and psychiatric disorders. Lancet Psychiatry. (2020) 7:628–37. doi: 10.1016/s2215-0366(20)30136-x 32563308

[3] Benca RM Obermeyer WH Thisted RA Gillin JC . Sleep and psychiatric disorders. A meta-analysis. Arch Gen Psychiatry. (1992) 49:651–68. doi: 10.1001/archpsyc 1386215

[4] Babson KA Trainor CD Feldner MT Blumenthal H . A test of the effects of acute sleep deprivation on general and specific self-reported anxiety and depressive symptoms: an experimental extension. J Behav Ther Exp Psychiatry. (2010) 41:297–303. doi: 10.1016/j.jbtep.2010.02.008 20231014 PMC2862829

[5] Reeve S Emsley R Sheaves B Freeman D . Disrupting sleep: The effects of sleep loss on psychotic experiences tested in an experimental study with mediation analysis. Schizophr Bull. (2018) 44:662–71. doi: 10.1093/schbul/sbx103 PMC5890488 28981834

[6] Palmer CA Alfano CA . Sleep and emotion regulation: An organizing, integrative review. Sleep Med Rev. (2017) 31:6–16. doi: 10.1016/j.smrv.2015.12.006 26899742

[7] Tempesta D Couyoumdjian A Curcio G Moroni F Marzano C De Gennaro L . Lack of sleep affects the evaluation of emotional stimuli. Brain Res Bull. (2010) 82:104–8. doi: 10.1016/j.brainresbull.2010.01.014 20117179

[8] Cieza A Causey K Kamenov K Hanson SW Chatterji S Vos T . Global estimates of the need for rehabilitation based on the Global Burden of Disease study 2019: a systematic analysis for the Global Burden of Disease Study 2019. Lancet. (2021) 396:2006–17. doi: 10.1016/S0140-6736(20)32340-0 PMC7811204 33275908

[9] Wulff K Dijk DJ Middleton B Foster RG Joyce EM . Sleep and circadian rhythm disruption in schizophrenia. Br J Psychiatry. (2012) 200:308–16. doi: 10.1192/bjp.bp.111.096321 22194182 PMC3317037

[10] Foster RG Peirson SN Wulff K Winnebeck E Vetter C Roenneberg T . Sleep and circadian rhythm disruption in social jetlag and mental illness. Prog Mol Biol Transl Sci. (2013) 119:325–46. doi: 10.1016/b978-0-12-396971-2.00011-7 23899602

[11] Waite F Evans N Myers E Startup H Lister R Harvey AG . The patient experience of sleep problems and their treatment in the context of current delusions and hallucinations. Psychol Psychotherapy: Theory Res Pract. (2016) 89:181–93. doi: 10.1111/papt.12073 PMC4879509 26285922

[12] Wee ZY Yong SWL Chew QH Guan C Lee TS Sim K . Actigraphy studies and clinical and biobehavioural correlates in schizophrenia: a systematic review. J Neural Transm (Vienna). (2019) 126:531–58. doi: 10.1007/s00702-019-01993-2 30888511

[13] Castelnovo A Graziano B Ferrarelli F D'Agostino A . Sleep spindles and slow waves in schizophrenia and related disorders: main findings, challenges and future perspectives. Eur J Neurosci. (2018) 48:2738–58. doi: 10.1111/ejn.13815 29280209

[14] Freeman D Stahl D McManus S Meltzer H Brugha T Wiles N . Insomnia, worry, anxiety and depression as predictors of the occurrence and persistence of paranoid thinking. Soc Psychiatry Psychiatr Epidemiol. (2012) 47:1195–203. doi: 10.1093/actrade/9780199567157.001.0001 21928153

[15] Sheaves B Bebbington PE Goodwin GM Harrison PJ Espie CA Foster RG . Insomnia and hallucinations in the general population: Findings from the 2000 and 2007 British Psychiatric Morbidity Surveys. Psychiatry Res. (2016) 241:141–6. doi: 10.1016/j.psychres.2016.03.055 27173659 PMC4922385

[16] Reeve S Nickless A Sheaves B Freeman D . Insomnia, negative affect, and psychotic experiences: Modelling pathways over time in a clinical observational study. Psychiatry Res. (2018) 269:673–80. doi: 10.1016/j.psychres.2018.08.090 30216919 PMC6215774

[17] Kasanova Z Hajdúk M Thewissen V . Temporal associations between sleep quality and paranoia across the paranoia continuum: An experience sampling study. (2020) 129:122–30. doi: 10.1037/abn0000453 31343182

[18] Geoffroy PA Hoertel N Etain B Bellivier F Delorme R Limosin F . Insomnia and hypersomnia in major depressive episode: Prevalence, sociodemographic characteristics and psychiatric comorbidity in a population-based study. J Affect Disord. (2018) 226:132–41. doi: 10.1016/j.jad.2017.09.032 28972930

[19] Palagini L Baglioni C Ciapparelli A Gemignani A Riemann D . REM sleep dysregulation in depression: state of the art. Sleep Med Rev. (2013) 17:377–90. doi: 10.1016/j.smrv.2012.11.001 23391633

[20] Arfken CL Joseph A Sandhu GR Roehrs T Douglass AB Boutros NN . The status of sleep abnormalities as a diagnostic test for major depressive disorder. J Affect Disord. (2014) 156:36–45. doi: 10.1016/j.jad.2013.12.007 24412322

[21] Geoffroy P Scott J Boudebesse C Lajnef M Henry C Leboyer M . Sleep in patients with remitted bipolar disorders: a meta‐analysis of actigraphy studies. Acta Psychiatrica Scandinavica. (2015) 131:89–99. doi: 10.1111/acps.12367 25430914

[22] Christiansen DM McCarthy MM Seeman MV . Where sex meets gender: How sex and gender come together to cause sex differences in mental illness. Front Psychiatry. (2022) 13:856436. doi: 10.3389/fpsyt.2022.856436 35836659 PMC9273892

[23] Green T Flash S Reiss AL . Sex differences in psychiatric disorders: what we can learn from sex chromosome aneuploidies. Neuropsychopharmacology. (2019) 44:9–21. doi: 10.1038/s41386-018-0153-2 PMC6235860 30127341

[24] Martini MI Kuja-Halkola R Butwicka A Du Rietz E D’Onofrio BM Happé F . Sex differences in mental health problems and psychiatric hospitalization in autistic young adults. JAMA Psychiatry. (2022) 79:1188–98. doi: 10.1001/jamapsychiatry.2022.3475 36287538 PMC9607919

[25] McGrath JJ Al-Hamzawi A Alonso J Altwaijri Y Andrade LH Bromet EJ . Age of onset and cumulative risk of mental disorders: a cross-national analysis of population surveys from 29 countries. Lancet Psychiatry. (2023) 10:668–81. doi: 10.1016/s2215-0366(23)00193-1 37531964 PMC10529120

[26] Seeman MV Lang M . The role of estrogens in schizophrenia gender differences. Schizophr Bull. (1990) 16:185. doi: 10.1093/schbul/16.2.185 2197713

[27] Searles S Makarewicz JA Dumas JA . The role of estradiol in schizophrenia diagnosis and symptoms in postmenopausal women. Schizophr Res Treat. (2018) 196:35–8. doi: 10.1016/j.schres.2017.05.024 28587815

[28] Hantsoo L Jagodnik KM Novick AM Baweja R di Scalea TL Ozerdem A . The role of the hypothalamic-pituitary-adrenal axis in depression across the female reproductive lifecycle: current knowledge and future directions. Front Endocrinol. (2023) 14:1295261. doi: 10.3389/fendo.2023.1295261 38149098 PMC10750128

[29] Murray G Harvey A . Circadian rhythms and sleep in bipolar disorder. Bipolar Disord. (2010) 12:459–72. doi: 10.1002/9780470661277.ch20 20712747

[30] Takaesu Y . Circadian rhythm in bipolar disorder: a review of the literature. Psychiatry Clin Neurosci. (2018) 72:673–82. doi: 10.1111/pcn.12688 29869403

[31] Xiu M Li W Lan Y Li K Lang X . Differentiation across bipolar disorder and major depressive disorder by whole-night polysomnographic findings. J Clin Psychiatry. (2024) 85:23m15210. doi: 10.4088/jcp.23m15210 39145682

[32] Iber C Ancoli-Israel S Chesson AL . The AASM manual for the scoring of sleep and associated events: rules, terminology and technical specifications. Am Acad Sleep Medicine Westchester. (2007).

[33] Boulos MI Jairam T Kendzerska T Im J Mekhael A Murray BJ . Normal polysomnography parameters in healthy adults: a systematic review and meta-analysis. Lancet Respir Med. (2019) 7:533–43. doi: 10.1016/s2213-2600(19)30057-8 31006560

[34] Burgard SA Ailshire JA . Gender and time for sleep among U.S. adults. Am Sociol Rev. (2013) 78:51–69. doi: 10.1177/0003122412472048 25237206 PMC4164903

[35] Chan MS Chung KF Yung KP Yeung WF . Sleep in schizophrenia: A systematic review and meta-analysis of polysomnographic findings in case-control studies. Sleep Med Rev. (2017) 32:69–85. doi: 10.1016/j.smrv.2016.03.001 27061476

[36] Wang Y-Q Li R Zhang M-Q Zhang Z Qu W-M Huang Z-L . The neurobiological mechanisms and treatments of REM sleep disturbances in depression. Curr Neuropharmacol. (2015) 13:543–53. doi: 10.2174/1570159x13666150310002540 26412074 PMC4790401

[37] Vreeburg SA Hoogendijk WJ van Pelt J DeRijk RH Verhagen JC van Dyck R . Major depressive disorder and hypothalamic-pituitary-adrenal axis activity: results from a large cohort study. Arch Gen Psychiatry. (2009) 66:617–26. doi: 10.1001/archgenpsychiatry.2009.50 19487626

[38] Plotsky PM Owens MJ Nemeroff CB . Psychoneuroendocrinology of depression: hypothalamic-pituitary-adrenal axis. Psychiatr Clinics North America. (1998) 21:293–307. doi: 10.1016/s0193-953x(05)70006-x 9670227

[39] Cipriani A Furukawa TA Salanti G Chaimani A Atkinson LZ Ogawa Y . Comparative efficacy and acceptability of 21 antidepressant drugs for the acute treatment of adults with major depressive disorder: a systematic review and network meta-analysis. Lancet. (2018) 391:1357–66. doi: 10.1176/appi.focus.16407 29477251 PMC5889788

